# A Manual of Surgical Treatment

**Published:** 1899-12

**Authors:** 


**A Manual of Surgical Treatment.** By W. Watson Cheyne, M.B., F.R.S., and F. F. Burghard, M.D. Part I. Pp. xiv., 285. London: Longmans, Green and Co. 1899.

A thorough knowledge of the nature of a disease we have to treat is essential, but our great aim is always to treat the patient in the best possible way. It is so easy to read of the treatment of a case in a text-book of surgery, but it is very difficult when we have to treat a case to determine which of several methods—all recommended by competent surgeons—shall be employed, or even whether there is any real evidence to show that any method of treatment is of value. It is not so much to assist us in deciding which plan of treatment to choose that the authors have written this work, as to give us the full details of the methods which they would themselves employ. This is very important. When we are engaged in active practice we need minute directions to which the mere student would pay but little heed.

In this volume those subjects are considered which would occupy the early chapters of a text-book of surgery—Inflammation, Abscess, Syphilis, Tumours, &c., &c. Dr. Silk contributes a very able article on the administration of anæsthetics. As an instance of the way in which information is given as to details, we would refer to the directions for the electrolysis of nævi. In the treatment of wounds we have a very trustworthy guide to antiseptic surgery. Many of the chapters are like those by Mr. Watson Cheyne in Treves's *System of Surgery*, only the question of treatment receives so much more attention and the details of the methods advised are given at length.

We heartily commend this book, and feel sure that the possession of the work in its complete form of six volumes will be of very great value to the busy general practitioner, as well as a help to the surgical specialist. The book is dedicated to Lord Lister, and there could not be a more fitting dedication, for, as all who know Mr. Watson Cheyne's work will readily believe, the methods recommended by the author are just those which the application of the great Listerian principles would suggest.

